# Correction to: ‘The ethics of genome editing in non-human animals: a systematic review of reasons reported in the academic literature’ (2019) by de Graeff *et al.*

**DOI:** 10.1098/rstb.2023.0202

**Published:** 2023-09-25

**Authors:** Nienke de Graeff, Karin R. Jongsma, Josephine Johnston, Sarah Hartley, Annelien L. Bredenoord

*Phil. Trans. R. Soc. B* **374**, 20180106. (Published online 25 May 2019) (https://doi.org/10.1098/rstb.2018.0106)

Errors in some of the references in the text and in Appendix A, table 3 were identified after publication.

The following corrections have been made in the published article. Note that page numbers refer to the PDF version.

Page 4. ‘Fourth, genome editing could help to meet the challenge of producing more food more sustainably to ensure that the future human population can be fed [34,78,79,141,142]…’—an additional reference 143 was added.

Page 5. ‘Finally, gene drives could be a cost-effective strategy for controlling the transmission of vector-borne diseases [6,27,109]’—reference 109 has been corrected to 102.

Page 5. ‘The potential for off-target mutations affecting the gene drive was mentioned as another risk [7,20,35,83]…’—an additional reference 143 was added.

Page 7. ‘Authors also mentioned that genome editing could result in off-target mutations or unintended effects, which could negatively affect animal health [9,80,90,104,139].’—an additional reference 143 was added.

Page 7. ‘Second, it was argued that genome editing could be an affront to an animal's dignity [96]…’—an additional reference 36 was added.

Appendix A, table 3. Several changes were made as outlined below. The full corrected table is shown below.

Page 11.

‘could facilitate xenotransplantation, which could be a solution to the human donor shortage’—*n* was changed from 26 to 36 and the list of references was changed from [25,37,44,45,48,59–73,94,105,108,111,133,139] to [26,37,39,44, 45,47,48,50,51,60,62–77,93,94,105,108,111,130,133,135,139,140]. This line moved to the top of the table.

Page 12.

Some incorrect formatting was amended (some information was appearing in the wrong columns).

‘could lead to advances in scientific understanding or technological advances’—the list of references was changed from [12,29,64,72,79,102, 126,131,140] to [12,21,26,33,69,75,81,102,134].

‘could be relatively inexpensive in comparison to previous approaches’—the list of references was changed from [9,17,50,102,108,124,130,133,134] to [9,20,23,26,34,110,114,132,142].

‘could save costs for the farming industry’—the list of references was changed from [23,65,72,76,83,84,97,125,141] to [19,70,75,79,86,87,96,98,126].

‘could accelerate and/or enhance the trait improvement currently accomplished by classic breeding’—the list of references was changed from [10,45,47,76,96,98,108,130] to [10,47,79,85,94,99,140,142].

‘could increase production efficiency’—the list of references was changed from [17,25,46,48,65,82,97] to [19,48,70,96, 115,133,139].

Page 13.

Some incorrect formatting was amended (some information was appearing in the wrong columns).

‘could still have inadequate gene targeting efficiency, off-target effects or cause mosaic mutations’—*n* was changed to 11 and an additional reference [76] added.

Page 16.

‘could reduce the numbers of animals used to create model organisms compared to traditional methods, which typically sacrifice many animals before achieving the desired genotype and phenotype’—this title was changed to ‘could be used to decrease the suffering of research animals’, *n* was changed to 2 and an additional reference [53] added.

**Appendix A. Table 3.** Reasons for and against the development and/or use of genome editing technologies in animals.

		*n*	references	technologies
***human-related reasons***				
*human health*				
*for* (*n* = 8)	could facilitate xenotransplantation, which could be a solution to the human donor shortage	36	[26,37,39,44,45,47,48,50,51,60,62–77,93,94, 105,108,111,130,133,135,139,140]	various (ZFN, TALEN, CRISPR)
	could enhance research by creating better animal model systems of human disease	35	[3,4,7,11,19,26,34–51,92–95,108,128,129,133,138–140]	various (ZFN, TALEN, CRISPR); genome editing
	could improve human health by reducing the burden of vector-borne diseases such as malaria	35	[5,6,19–33,52,101–109,114,124–127,135,139,140,143]	gene drives; genetic modification; genome editing
	could expedite research in other species, including non-human primates, which provide more accurate models for human (neurological) disease	12	[36,43,55–63,126]	CRISPR
	could help to meet the challenge of producing more food more sustainably to ensure the future global population can be fed	6	[34,78,79,141–143]	various (ZFN, TALEN); genetic modification
	could improve human health through the provision of new medicines and therapies	4	[26,126,133,140]	various (TALEN, CRISPR)
	could enable genome engineering in non-human primates; this could be considered ethically problematic, but it is much more ethically problematic to watch people die who could be saved	1	[57]	CRISPR
	could be used to create a chicken strain with low allergenicity	1	[126]	CRISPR
*against* (*n* = 8)	re simplifying and speeding up the production of new transgenic animal models of human disease: most of such models fail to directly benefit humans; this lack of reproducibility may put human research participants at risk at a later stage	3	[53,54,110]	CRISPR
	re bringing routine genome engineering of non-human primates within reach, which could help identify genetic underpinnings of disease or develop therapies: the moral permissibility of this approach is questionable given available alternatives	2	[110,126]	CRISPR
	could pose risks to human health if genetic modification is not successful in creating mosquitoes resistant to infections, but instead confers no resistance or actually reduces resistance to the target infection	2	[102,109]	genetic modification; gene drives
	could disrupt ecosystems, which could be harmful to human populations that depend on them	2	[20,143]	gene drives
	could be used to re-create species that may become a vector or reservoir for viruses that can be harmful for human beings	1	[81]	genetic engineering
	re use to create a chicken strain with low allergenicity: there may not be a compelling need for doing so because allergy usually only occurs in children, and alternatives and egg substitutes are available	1	[80]	various (ZFN, TALEN, CRISPR)
	re use to produce better quality food: little is known about the effects these modified organisms would have on humans when consumed	1	[35]	CRISPR
	could increase productivity of the livestock sector: this is an undesirable outcome given the negative impact of meat consumption on human health	1	[104]	various (ZFN, TALEN, CRISPR)
*efficiency*				
*for* (*n*=14)	could be more efficient, versatile, precise, easy to use or accurate than previous editing technologies	39	[3,4,6,7,9,33–35,37,40–43,46,49–51,53,56,63,64,69,75,79,82–84,98,105,110,114,126,129,131,133,134,136,140,142]	various (ZFN, TALEN, CRISPR)
	could lead to advances in scientific understanding or technological advances	9	[12,21,26,33,69,75,81,102,134]	various (ZFN, TALEN, CRISPR; genetic modification; active genetics^a^)
	could be relatively inexpensive in comparison to previous approaches	9	[9,20,23,26,34,110,114,132,142]	CRISPR, gene drives
	could save costs for the farming industry	9	[19,70,75,79,86,87,96,98,126]	various (ZFNs, TALEN, CRISPR)
	could accelerate and/or enhance the trait improvement currently accomplished by classic breeding	8	[10,47,79,85,94,99,140,142]	various (ZFN, TALEN, CRISPR, gene drives)
	could increase production efficiency	7	[19,48,70,96,115,133,139]	various (ZFNs, TALEN, CRISPR); genetic modification
	could be a potentially efficient and rapid tool to improve important traits in livestock	3	[26,96,97]	various (ZFNs, TALEN, CRISPR)
	could be a cost-effective strategy to control the transmission of vector-borne diseases	3	[6,27,102]	genetic modification, gene drives
	could increase economic productivity in animals bred for human consumption	2	[97,137]	various (ZFNs, TALEN, CRISPR); genetic engineering
	could provide animals with disease resistance, which could reduce the overuse of antibiotics	2	[98,139]	various (TALEN, CRISPR)
	could be used to eradicate vector-borne diseases in a more efficacious and/or logistically less complex way than other efforts to eliminate these diseases	2	[27,28]	gene drives
	could be used for pest control, being more precise or effective than other pest management methods such as pesticides	2	[20,109]	gene drives
	re the possibility of off-target effects: these are fewer and more controlled compared with the mutations that are caused by generally accepted technologies such as conventional breeding	2	[80,142]	various (ZFNs, TALEN, CRISPR)
	re the possibility of off-target effects: these can be minimized by careful design and testing, and their effects are largely identical to those of the natural processes that continually create variation in the genomes of food animals	1	[85]	various (ZFNs, TALEN, CRISPR)
*against* (*n* = 1)	could still have inadequate gene targeting efficiency, off-target effects or cause mosaic mutations	11	[42,47,54,55,58–60,63,76,95,116]	various (ZFN, TALEN, CRISPR)
*risks and uncertainty*				
*against* (*n* = 8)	could spread beyond its target population owing to accidental release, cross-breeding or gene flow; this release could have unpredictable ecological consequences	12	[20,23,28,35,82,83,88,89,106,109,124,143]	gene drives
	could introduce off-target mutations into the gene pool and spread these across a species	5	[7,20,35,83,143]	gene drives
	could have novel features that are unprecedented and unexpected, so the risks and consequences are difficult or even impossible to characterize beforehand	4	[20,102,117,143]	various (synthetic biology, gene drives, genetic modification)
	could involve risks of deliberate release of (disease carrying [23]) genetically modified mosquitoes to the environment	2	[105,117]	synthetic biology (including genome editing), gene drives
	could be used to serve the (economic) interests of particular groups with little concern for the general interest	2	[20,115]	various (ZFNs, TALEN, CRISPR, gene drives)
	could have unexpected effects because our knowledge and understanding of the genetic background of complex traits is incomplete	1	[96]	various (ZFNs, TALEN, CRISPR)
	could have non-negligible risks because breaches of containment are impossible to rule out and, once released, just a few escaped genetically modified mosquitoes could be capable of spreading transgenes on a global scale	1	[22]	gene drives
	could benefit humans if used for applications to human disease and agricultural production, however these applications could primarily benefit the current generation, with secondary benefits and potential risks placed upon future generations	1	[143]	gene drives
*for* (*n* = 7)	re the potential to spread beyond its target population or have unintended consequences: various designs of the gene drive and other containment measures may mitigate these risks	13	[5,19,23,25,26,31,32,88,89,102,105,125,131]	gene drives
	re novel features: could be considered similar to conventional breeding owing to the similarity to natural mutations and the absence of transgenes	4	[47,48,80,85]	various (ZFNs, TALEN, CRISPR)
	re potential risks: could be researched in a phased approach, allowing sufficient time to evaluate the efficacy and safety of gene drives before regulatory decisions are made on whether they will be suitable for use	2	[32,135]	gene drives
	re potential for off-target effects with negative effects: genome modification is more precise and consequently has far fewer risks than conventional breeding	1	[79]	various (ZFN, TALEN)
	re potential risks: it is generally more difficult to prove that something is safe than to find potential risks; the damage of not using a new technique may exceed its potential risks	1	[96]	various (ZFNs, TALEN, CRISPR)
	re uncertain consequences: these are not in themselves a sufficient reason not to use the technology; the magnitude and likelihood of these risks ought to be thoroughly analysed and balanced against the potential benefits	1	[101]	gene drives
	re potential risks: these ought to be balanced with the risks and harm caused by the unmodified wild-type	1	[23]	gene drives
*public acceptability*				
*for* (*n* = *6*)	could be more acceptable to the public than previous technologies, as no foreign DNA is introduced	4	[9,33,96,97]	various (ZFNs, TALEN, CRISPR)
	could increase the chance of a publicly justified policy permitting genome editing	1	[9]	CRISPR
	could be less controversial than using pesticides for pest control	1	[20]	gene drives
	could impact community members who have not consented to the release of genetically modified mosquitoes; however, this may be justifiable if the public health benefits of the trial for the community are important enough	1	[102]	genetic modification
	could be used in field trials with genetically modified animals while respecting the interests of community members if community advisory boards and a community authorization process are used	1	[107]	gene drives
	re public resistance: could lead to resistance when modified mosquitoes cross borders to communities who did not agree with this; however, various designs of the gene drive may prevent this, enabling local communities to make local decisions	1	[31]	gene drives
*against* (*n* = 1)	could lead to public resistance	6	[12,22,89,102,108,126]	gene drives; genetic modification; genome editing, genetic engineering
***animal-related reasons***				
*animal welfare*				
*for* (*n* = 13)	could decrease animal suffering in dairy farming by creating dehorned cattle, preventing invasive and painful dehorning	10	[9,19,78–80,85,96,126,139,140]	various (ZFNs, TALEN, CRISPR)
	could counter welfare problems by creating the so-called diminished animals in which the ability to sense pain is impaired	8	[78,112,115,116,119–121,137]	genome editing (CRISPR); genetic engineering/modification
	could increase animal health and welfare by providing animals with disease resistance	8	[78,80,96,98,126,133,135,139]	various (ZFNs, TALEN, CRISPR)
	could increase adaptations to different environmental conditions	2	[19,137]	various (ZFNs, TALEN, CRISPR); genetic engineering
	could be used to prevent the killing of day-old male chicks	2	[100,126]	CRISPR; genetic modification
	re the possible creation of animals with welfare problems: if they have a life worth living we cannot say that they are worse off owing to the genetic modification, for if they had not been created with genetic modification, they would not have existed at all	2	[115,118]	genetic modification
	could be used to decrease the suffering of research animals	2	[53,110]	CRISPR
	re off-target effects: could result in fewer off-target effects than previous techniques, which could improve welfare of genetically modified animals	1	[9]	CRISPR
	could remove known harmful recessive alleles that impair fertility or health and in that sense repair accumulated damage in the genome of breeding animals	1	[96]	various (ZFNs, TALEN, CRISPR)
	could prevent wild animal suffering by using genome editing to change reproductive behaviour; the harm that would be prevented by doing so would outweigh the harm of developing and testing these strategies	1	[114]	CRISPR
	could lead us to ignore the predicament of the animal and to accept negative effects on animal welfare for the sake of other goals; however, this concern may be addressed by using less drastic gene drive designs and using these to promote animal welfare	1	[9]	gene drives
	re applications that would permit even greater intensification of farming resulting in decreased animal welfare: this seems unlikely given recent trends of companies to improve animal welfare	1	[78]	various (ZFNs, TALEN, CRISPR)
	could be a humane method to eliminate invasive species	1	[6]	gene drives
*against* (*n* = 11)	could result in off-target mutations or unintended effects, which could negatively affect animal health	6	[9,80,90,104,139,143]	various (ZFNs, TALEN, CRISPR)
	could contribute to animal suffering by perpetuating the use of animals in research	5	[9,36,53,108,110]	genome editing; CRISPR
	could result in secondary complications that are bad for animal welfare (e.g. increased muscle growth could lead to increased rates of caesarean sections, leg problems or breathing complications)	3	[80,96,104]	various (ZFNs, TALEN, CRISPR)
	could be used for applications that would permit even greater intensification of farming; this outcome would be undesirable	3	[36,104,116]	various (ZFNs, TALEN, CRISPR); genome engineering
	could be used to the decrease animal suffering (by creating polled cattle or diminished animals); however, there are alternatives to doing so (e.g. by improving animals’ environments)	2	[80,118]	various (ZFNs, TALEN, CRISPR)
	could be combined with somatic cell nuclear transfer (SCNT) cloning to deliver the nuclease-mediated genetic alterations, which are associated with embryonic losses, postnatal death and birth defects	2	[95,97]	various (ZFNs, TALEN, CRISPR)
	could bring routine genome editing of non-human primates within reach; this use of the technologies may substantially diminish these organisms' welfare and quality of life	1	[110]	CRISPR
	re use to prevent wild animal suffering: the complexity of ecosystems, the unpredictability of climate change and the indeterminacy of human behaviour leave us with too little confidence that this aim will be successful	1	[122]	genome editing
	re use to create diminished animals that lack the affective dimension of pain: no proof of concept experiment has been done on farm animals and conducting these experiments would itself cause suffering	1	[116]	genetic engineering
	re use to revive extinct species: the revived animals may end up suffering either as a result of the processes used or because of their particular genomic variations	1	[12]	genetic engineering
	re use to revive extinct species: the re-created species may become a vector or reservoir for viruses that can be harmful for other animals	1	[81]	genetic engineering
*animal dignity and species-specific characteristics*				
*against* (*n* = 9)	could be objectionable because it instrumentalizes animals by using them as mere objects to serve human purposes	5	[36,64,81,104,115]	various (ZFNs, TALEN, CRISPR)
	could be used to revive extinct species or create gene-edited pets, but it is questionable if physiological limits should be altered or animals should be exploited for unimportant human purposes like entertainment	3	[12,126,128]	various (TALEN, CRISPR)
	could impinge on animals' dignity as altering the genome of an animal is a failure to acknowledge its dignity or prevents the animal from living according to its instinct	3	[36,96,111]	various (ZFNs, TALEN, CRISPR)
	could affect the ‘telos’ (the essence and purpose of a creature) if they are genetically altered to the point where they lose the behaviour that characterizes that animal	3	[80,120,137]	various (ZFNs, TALEN, CRISPR); genetic modification
	could expedite transgenesis in other species, including non-human primates, which probably occupy a level of moral status that would obligate us to protect them from being used in this way or to allow it only in extremely exceptional circumstances	2	[53,110]	CRISPR
	could create diminished animals to decrease animal suffering, but this is an inappropriate response to the historical wronging of agricultural animals; we have a duty to repair these wrongs	2	[116,119]	genome editing (CRISPR mentioned)
	could only be rightfully done if the permissibility of genome editing in research is evaluated for each species on its own merits	1	[36]	CRISPR
	could be used to facilitate xenotransplantation, which could be considered ethically untenable as it compromises species boundaries	1	[64]	CRISPR
	could be viewed as the initiation of increasingly imbalanced power distribution between humans and animals	1	[80]	various (ZFNs, TALEN, CRISPR)
*for* (*n* = 11)	re breaching species norms if used for animal diminishment: species norms are only indirectly morally significant, as a generally useful guide to evaluating animal welfare	2	[118,119]	genome editing (CRISPR)
	re violating animal dignity or integrity: such arguments focus only on respect for individual animals, they ultimately cannot justify an objection that is based on a species-norm, as is the case in the discussion on enhancement	2	[115,118]	genetic modification
	re use to create diminished animals, which could be said to harm these animals as their species-typical essence would be changed: as the literature about human disability has taught us, we should not assume that ‘disabilities’ caused by diminishment make animals worse off	2	[78,112]	various (ZFNs, TALEN, CRISPR; genetic engineering)
	re violating rights, violating dignity or wrongly instrumentalizing: genome editing determines which individual will come into existence rather than modifying existing individuals, making it hard to say how its rights could have been infringed, its dignity violated, or even that it has been wrongly instrumentalized	1	[119]	genome editing (CRISPR mentioned)
	re breaching the sanctity of the lives of mosquitoes by making them go extinct: neither existing mosquitoes nor the species holistically bear a significant degree of moral status	1	[101]	gene drives
	re impinging on an animal's dignity by making them serve better as objects for human use: the Kantian concept of dignity cannot be applied to animals, for this concept is tied to prerequisite conditions that animals do not possess	1	[113]	genetic engineering
	re use to modify an animal's telos or nature: this could be morally acceptable if the animals are made less miserable or happier as one does not morally wrong the telos by changing it; only individuals can be wronged	1	[121]	genetic engineering
	could be used to prevent additional violations to animal rights, which would be preferable to the status quo, even on an account that considers raising animals for human consumption impermissible	1	[78]	various (ZFNs, TALEN, CRISPR)
	re impinging on an animal's integrity or dignity and thereby harming it even if welfare is improved: what is good for an individual must in some way resonate with that individual; what is good for it cannot diverge from its welfare	1	[78]	various (ZFNs, TALEN, CRISPR)
	re impact on the ‘telos’ of an animal: the animal's telos can still be respected if it is provided with an environment that fits its altered genetic predispositions	1	[78]	various (ZFNs, TALEN, CRISPR)
	re impact on the ‘telos’ of an animal: the idea that there is some ‘true essence’ of a species is mistaken as behaviours and tendencies change over time, making it hard to see why this should be seen as morally problematic	1	[78]	various (ZFNs, TALEN, CRISPR)
***environment-related reasons***				
*environmental considerations*				
*against* (*n* =10)	could have unknown negative effects on ecosystems	13	[6,7,20,28,34,35,82,83,106,108,126,139,143]	gene drives
	could cross moral limits by exceeding the extent to which humans breach natural boundaries or act out of hubris; nature/life cannot be completely manufactured or planned and we ought to acknowledge their unpredictability	4	[12,36,115,117]	various (ZFNs, TALEN, CRISPR, synthetic biology)
	could constitute an unnatural interference with nature	2	[100,115]	various (ZFNs, TALEN, CRISPR); genetic modification
	could be used to revive extinct species, for which there may no longer be a niche	2	[12,81]	genetic engineering
	could be used to revive extinct species, which might diminish the desire to protect existing species	2	[12,81]	genetic engineering
	re use to revive extinct species: genome editing will fail to genuinely recreate species while preserving their species identity	2	[12,123]	genome editing; genetic engineering
	could disrupt the natural order; although this order should not hold an intrinsic moral value, deleting genetic diversity could carry risks by deleting traits that are advantageous	1	[105]	gene drives
	could lead to increased productivity of the livestock section, which is not desirable given the negative impact of this sector on the environment (e.g. greenhouse gas production and water and land pollution)	1	[104]	various (ZFNs, TALEN, CRISPR)
	could be used to control certain invasive species; if this succeeds, this could become a trojan horse to legitimate the eradication of other species without questioning to whom or what they are harmful	1	[20]	CRISPR, gene drives
	could be more transformative, uncontrollable and ecologically damaging than organisms modified to contain self-limiting genes	1	[107]	gene drives
*for* (*n* = 9)	could enable ecological conservation by eradicating invasive species or reviving extinct species	8	[5,12,21,31,91,103,124,143]	active genetics^a^; gene drives; genetic engineering
	could help to develop and support more sustainable agricultural models	4	[5,31,32,105]	gene drives
	re potential to be considered unnatural or alike ‘playing God’: it is unclear what is meant by naturalness; furthermore, there is no reason to accept that the natural is necessarily good and the unnatural necessarily bad	2	[78,111]	various (ZFNs, TALEN, CRISPR); genetic engineering
	could contribute to reducing the environmental impact of animal production	1	[96]	various (ZFNs, TALEN, CRISPR)
	could protect threatened species and reduce invasive species to conserve the natural and cultural world for future generations, which could be imperative from an intergenerational justice perspective	1	[143]	gene drives
	re potential of driving mosquitoes to extinction being considered ‘playing god’ or displaying hubris: there may be sufficient reasons—such as saving many lives—that may justify improving the given	1	[101]	gene drives
	could be used to control agricultural pests; this may be a more environmentally sound control method than using insecticides	1	[23]	gene drives
	could be used to revive extinct species, which would be just; because humans killed extinct species and have the power to revive them, there is a duty to do so	1	[12]	genetic engineering
	re ecological risks created by using gene drives to prevent wild animal suffering by using genome editing to change reproductive behaviour: these risks may be offset by modifying other features of the ecosystem, too	1	[114]	CRISPR, gene drives

^a^Genetic manipulations in which a ‘genetic element is copied from one chromosome to the identical insertion site on the sister chromosome using cas9 and guide RNA elements’ [21].

